# Static and Dynamic Measurements of Compliance and Driving Pressure: A Pilot Study

**DOI:** 10.3389/fphys.2022.773010

**Published:** 2022-02-04

**Authors:** Pierre Tawfik, Muhammad K. Hayat Syed, Firas S. Elmufdi, Michael D. Evans, David J. Dries, John J. Marini

**Affiliations:** ^1^Pulmonary, Allergy, Critical Care, and Sleep Medicine, University of Minnesota, Minneapolis, MN, United States; ^2^Pulmonary and Critical Care Medicine, Baylor College of Medicine, Houston, TX, United States; ^3^Department of Medicine, Regions Hospital, Saint Paul, MN, United States; ^4^Clinical and Translational Science Institute, University of Minnesota, Minneapolis, MN, United States; ^5^Department of Surgery, Regions Hospital, Saint Paul, MN, United States; ^6^Department of Critical Care and Acute Care Surgery, University of Minnesota, Minneapolis, MN, United States

**Keywords:** compliance, static compliance, dynamic compliance, driving pressure, plateau pressure, respiratory mechanics, ventilator induced lung injury, mechanical ventilation

## Abstract

**Rationale:**

Monitoring tidal cycle mechanics is key to lung protection. For this purpose, compliance and driving pressure of the respiratory system are often measured clinically using the plateau pressure, obtained after imposing an extended end-inspiratory pause, which allows for relaxation of the respiratory system and redistribution of inflation volume (method A). Alternative methods for estimating compliance and driving pressure utilize the measured pressure at the earliest instance of zero flow (method B), the inspiratory slope of the pressure-time tracing during inflation with constant flow (method C), and the expiratory time constant (method D).

**Methods:**

Ten passive mechanically ventilated subjects, at a large tertiary referral center, underwent measurements of compliance and driving pressure using the four different methods. The inspiratory tidal volume, inspiratory to expiratory ratio, and positive end expiratory pressures were then adjusted from baseline and the measurements re-obtained.

**Results:**

Method A yielded consistently higher compliance and lower driving pressure calculations compared to methods B and C. Methods B and C most closely approximated one another. Method D did not yield a consistent reliable pattern.

**Conclusion:**

Static measurements of compliance and driving pressure using the plateau pressure may underestimate the maximum pressure experienced by the most vulnerable lung units during dynamic inflation. Utilizing the pressure at zero flow as a static measurement, or the inspiratory slope as a dynamic measurement, may calculate a truer estimate of the maximum alveolar pressure that generates stress upon compromised lung units.

## Introduction

Measuring compliance of the respiratory system provides critical insight into the mechanics of the lungs and chest wall and the dynamics of breathing. Apart from characterizing the ease or difficulty of chest inflation, tidal compliance tracks the severity and progress of acute lung injury (ARDS). Moreover, compliance determines the driving pressure necessary to inflate the lungs with a specific tidal volume, a key indicator of the risk for ventilator-induced lung injury (VILI) (Amato et al., 2015; Goligher et al., 2021). In current clinical practice, compliance and driving pressure are commonly measured under passive conditions after an extended end-inspiratory pause is performed to obtain the stable plateau pressure (P_*plateau*_). While this average value for driving pressure measured under “stop flow” conditions has definite worth, is simple to obtain, and is universally measured, it may underestimate the maximum stress to which some lung units are exposed under the dynamic conditions of tidally inflating the mechanically heterogeneous lungs of ARDS.

**FIGURE 1:**
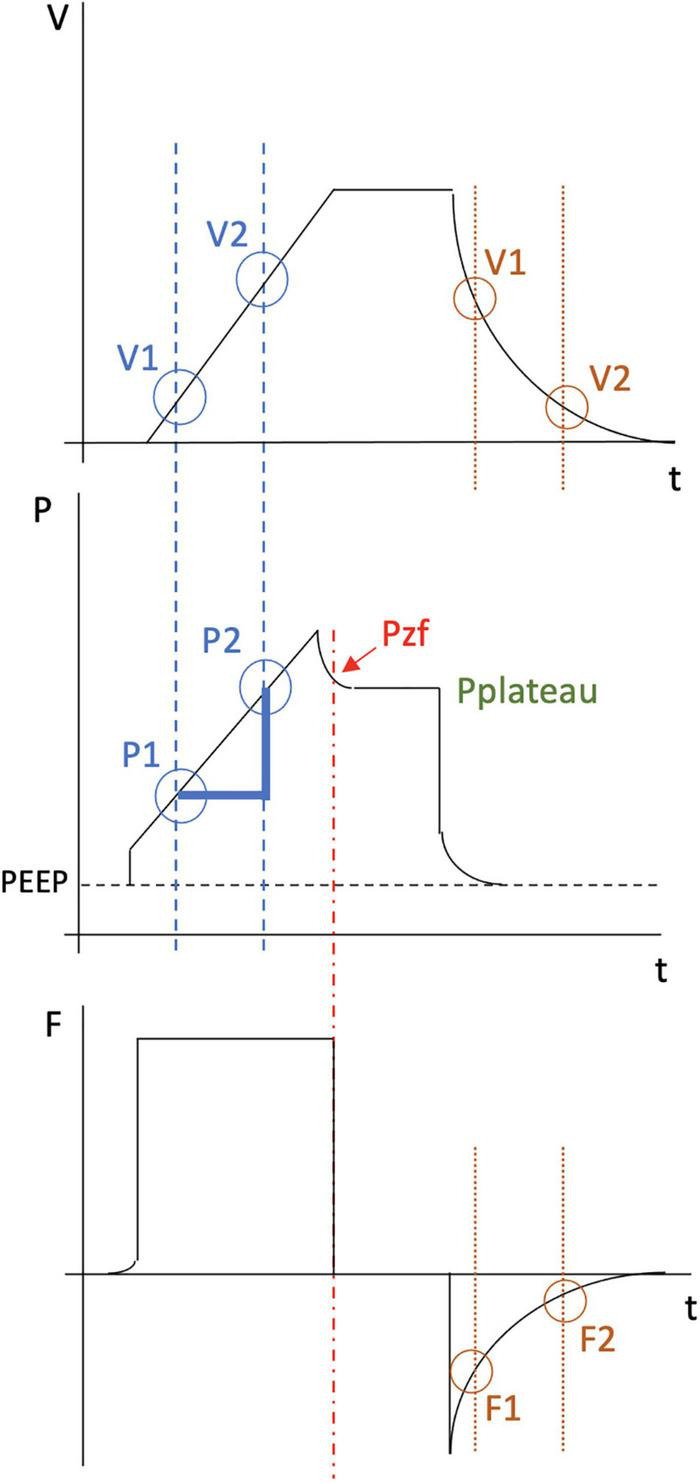
Illustration of the inspiratory and expiratory phase of a typical breath under constant flow and the various methods for calculating compliance and driving pressure. The top waveform plots volume (V) over time (t). The middle waveform plots pressure (P) over time. The bottom waveform plots flow (F) over time. Compliance calculation in method A (green) is performed by dividing the change in volume by the change in pressure (Pplateau minus PEEP). Compliance calculation in method B (red) is performed by dividing the change in volume by the change in pressure utilizing instead the pressure at zero flow (Pzf minus PEEP). Compliance calculation in method C (blue) is performed using the following equation: [V2-V1] / [P2-P1]. Compliance calculation in method D (orange) requires measurement of multiple expiratory time constants. For example, time constant #1 equals V1/F1. Time constant #2 equals V2/F2. Compliance is then calculated by dividing the average of the different time constants by the resistance. For each of these four methods, Driving Pressure = inspiratory volume / compliance.

When VILI prevention is the focus of attention, P_*plateau*_ (Method A) may not precisely reflect the maximum alveolar pressure experienced by the most vulnerable alveoli (Milic-Emili et al., 1990). The end inspiratory breath hold allows for relaxation of the respiratory system and redistribution of inflation volume and distending pressures. These processes usually decrease the measured pressure. An alternative method of measuring compliance uses the pressure measured at the first point of zero flow during the end-inspiratory pause (P_*zf*_), which usually occurs about 2 s earlier than the P_*plateau*_ (Method B) (Figure 1) (Milic-Emili et al., 1990). This latter method excludes the airflow resistance component from the recorded pressure measurement without allowing full relaxation and gas redistribution within the respiratory system. Measuring compliance using P_*plateau*_ and P_*zf*_ may still not accurately characterize the maximum pressure experienced by some alveoli, especially in a mechanically complex environment; both are measurements made under quasi-static conditions that may or may not perfectly characterize the non-static (dynamic) tidal inflation of the lungs.

Compliance can also be measured using dynamic values obtained during inflation or deflation. One such method calculates compliance during constant flow, based on the slope of the pressure-time tracing (Method C). During inflation under constant flow conditions, time is a direct linear analog of volume. Two pressure values during inflation and their corresponding volumes can therefore be used to measure compliance using the following equation: Compliance = (V2-V1)/(P2-P1) where P1 and P2 are two pressure measurements during inflation while V1 and V2 are the corresponding tidal volumes occurring at those same time points. Another dynamic method of assessing compliance (Method D) assumes uniexponential decay of the tidal volume and computes the deflation time constant, the product of resistance and compliance (R × C) (Al-Rawas et al., 2013). Under the assumptions of uniexponential decay and unchanging resistance, the time constant is a quotient of volume at any point on the deflation curve and its corresponding instantaneous flow rate. The calculated time constant is then divided by resistance measured at end-inspiration under constant flow conditions to obtain compliance, i.e., Compliance = expiratory time constant/resistance. For each of these four methods, Driving Pressure = inspiratory volume/compliance.

Although a signature property of bedside lung mechanics used in decision making, various questions arise regarding compliance and driving pressure measurements. It is unclear how these four alternative methods compare to one another in the same patient. It is also unknown how these relationships are altered by varying the ventilator settings of tidal volume and inspiratory flow. Our purpose, therefore, was to compare such methods of measuring compliance and driving pressure in the clinical setting and to explore the impact of ventilator parameters on these methods. We hypothesized that differences would arise between the inspiratory versus expiratory methods of compliance and driving pressure estimation as well as between these values obtained by the dynamic versus static methods.

## Materials and Methods

### Study Design

This study was performed at Regions Hospital in St. Paul, Minnesota, and was approved by the Regions Hospital Institutional Review Board. We conducted a prospective cohort study of passive mechanically ventilated subjects. All subjects receiving mechanical ventilation in the medical intensive care unit were evaluated for inclusion. Subjects 18 years and older who were deemed to be passively ventilated, either by receiving neuromuscular blockade, or who had a Richmond Agitation–Sedation Scale (RAAS) of −3 or −4 and demonstrated no evidence of active breathing at the bedside (i.e., over-breathing, dyssynchrony), were included. Subjects who were pregnant, had undergone prior lung, chest wall or abdominal surgeries, had known deformity of the chest wall, or had known parenchymal or obstructive lung disease were excluded. Family members of subjects meeting the study criteria were approached for consent.

### Study Procedures

Since deflation may not occur uniexponentially (Hamahata et al., 2020), there are potentially multiple time constants throughout expiration. For the purposes of this study, two time constants were measured, and their mean was used to obtain an average for the compliance equation.

After consent was obtained, subjects were re-evaluated at the bedside to ensure ongoing passive ventilation and stable hemodynamics. Prior to data collection, all subjects were ventilated using pressure regulated volume control. Each subject’s flow profile was changed to an equivalent constant flow, maintaining the subject’s same inspiratory to expiratory ratio (I:E), tidal volume (TV), respiratory rate (RR), oxygen concentration (FIO2) and positive end expiratory pressure (PEEP). At these “baseline” settings, an inspiratory breath hold was performed, followed by a breath without a hold maneuver, followed by a breath with an end expiratory hold. The ventilator screen was then frozen, and the cursor dial maneuvered to obtain the necessary measurements from the time-based tracings of pressure, volume, and flow. On the inspiratory limb of a pressure-volume display, two pressures and corresponding volumes were measured (for method C calculations). These points were chosen from the middle 80% of the inspiratory limb to avoid variability from inspiratory flow initiation and cessation. For the paused breaths, the P_*zf*_ was recorded, followed by the P_*plateau*_, measured 2 s after the breath hold was initiated (for methods A and B calculations). From the second breath (without a breath hold maneuver), two expiratory flow measurements and corresponding volume measurements were obtained from the middle 50% of the generated graph (method D). For method D, the initial 25% and latter 25% of the expiratory limb of the loop were excluded to best approximate an average time constant. Compliance and driving pressure were calculated for each method.

The following ventilator setting adjustments were then performed unless they violated the prespecified protocol safety parameters. Breath holds and data gathering steps were performed as detailed above for the following variations. Tidal volume was first increased from baseline and then decreased from baseline by 2 ml/kg of ideal body weight (IBW). Next, I:E ratio was increased from the baseline ratio by 1.0 and then decreased from baseline by 1.0. Finally, PEEP was increased and then decreased from its baseline value by 2 cmH2O. After each ventilator adjustment, we allowed for a 1-min equilibration period. The ventilator was not adjusted if the following pre-specified safety parameters were surpassed: tidal volume < 4 ml/kg of IBW or > 10 ml/kg of IBW; baseline P_*plateau*_ greater than 35 cmH2O; baseline PEEP < 5 cmH2O or PEEP > 12 cmH2O in a subject with BMI less than 30 kg/m^2^; baseline PEEP < 5 cmH2O or PEEP > 16 cmH2O in a subject with BMI greater than 30 kg/m^2^.

### Data Collection

Study data were collected and managed using Research Electronic Data Capture (REDCap) electronic data capture tools, a secure, web-based software platform, hosted at Regions Hospital (Harris et al., 2009).

### Statistical Analysis

Participants’ demographic and clinical characteristics were summarized using counts and rates or medians and ranges. Radar plots were used to visualize each participant’s compliance and driving pressure measurements at each ventilator setting for each of four calculation methods (Supplementary Materials 1, 2). To examine the average effect of ventilator setting changes on compliance and driving pressure for each calculation method, linear mixed-effects models were fit with fixed effects terms for tidal volume, I:E ratio, and total PEEP, and a random effect term for participant to account for repeated measurements within participant. To compare compliance and driving pressure between calculation methods at each ventilator setting combination, similar mixed-effects models were fit with the differences between each pair of calculation methods as the outcomes. Results are summarized using mean effects with 95% confidence intervals. Analyses were conducted using R version 4.1.1 (R Core Team, 2020).

## Results

**FIGURE 2:**
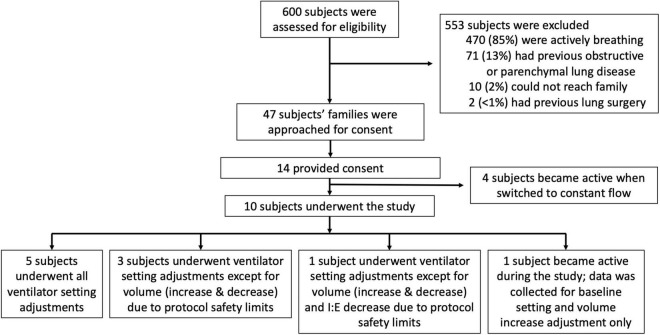
Flow diagram outlining subjects assessed for eligibility, reasons of exclusion or inability to enroll, and breakdown of the ventilator setting adjustments that were tested.

The study was conducted over 8 months from October 2020 through June 2021. The study was stopped by the investigators due to low enrollment, primarily owing to a majority of considered subjects having a degree of active breathing on the ventilator (Figure 2). Ten subjects completed the study (Table 1). Five subjects underwent all ventilator settings adjustments. The remainder completed some, but not all, of the adjustments due to protocol safety limits (Figure 2). The subjects were primarily male (7/10, 70%) and Caucasian (8/10, 80%). The median number of days undergoing mechanical ventilation at the time of data collection was three (range 1–10 days). Reasons for intubation were COVID ARDS (5/10, 50%), encephalopathy (2/10, 20%), cardiac arrest (1/10, 10%), cardiogenic shock (1/10, 10%), and aspiration pneumonia (1/10, 10%). P/F calculations indicated a range from normal oxygenation to all degrees of ARDS severity: median P/F of 134.5 (range 75–623). Of the five subjects who completed all ventilator setting adjustments, four subjects had moderate to severe ARDS by P/F criteria. A minority of subjects received epoprostenol (2/10, 20%) and a minority were prone at the time of data collection (3/10, 30%). These two interventions did not appear to create a discernable deviation or change in the compliance or driving pressure calculations. Only two subjects were paralyzed (2/10, 20%); lack of paralysis did not appear to create a pattern of deviation or inconsistency in the calculations.

**TABLE 1:** Demographics and clinical characteristics of the tested subjects.

Age (median, years)	61.5 (range 37–81)
**Sex**	
Male	7/10 (70%)
Female	3/10 (30%)
**Race**	
Caucasian	8/10 (80%)
Hispanic	1/10 (10%)
Southeast Asian	1/10 (10%)
Height (median, cm)	173.9 (range 154.9–188.0)
BMI (median, kg/m2)	32.3 (range 24.6–40.2)
Ventilator days at time of data collection (median, days)	3.0 (range 1–10)
**Primary diagnosis requiring intubation**	
Encephalopathy	2/10 (20%)
Aspiration pneumonia	1/10 (10%)
Cardiac arrest	1/10 (10%)
Cardiogenic shock	1/10 (10%)
COVID ARDS	5/10 (50%)
**RAAS**	
−3	2/10 (20%)
−4	4/10 (40%)
−5	4/10 (40%)
**Position at time of data collection**	
Supine, flat	1/10 (10%)
Supine, upright at 30–40 degrees	6/10 (60%)
Prone	3/10 (30%)
Baseline volume (median, mL)	444.5 (range 304–579)
Baseline I:E (median)	1:2.31 (range 1:1.47–1:3.29)
Baseline total PEEP (median, cmH2O)	8.57 (range 5–15)
pH (median)	7.29 (range 7.24–7.45)
PaO2/FIO2 (median, mmHg)	134.5 (range 75–623)
Hgb (median, g/dL)	9.0 (range 7.2–14.7)
WBC [median, ×10(9)/L]	11.3 (range 6.1–18.3)
**Number of subjects receiving each sedative**	
Hydromorphone	6/10 (60%)
Fentanyl	3/10 (30%)
Propofol	2/10 (10%)
Lorazepam	5/10 (50%)
Midazolam	4/10 (40%)
Dexmedetomidine	1/10 (10%)
Number of subjects receiving paralytics	2/10 (20%)
Number of subjects receiving inhaled epoprostenol	2/10 (20%)

**FIGURE 3:**
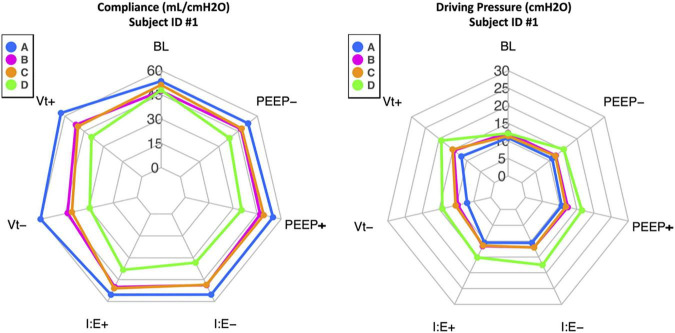
Example radar plots for one subject (ID #1) showing compliance (mL/cmH20) and driving pressure (cmH2O) calculations. The blue line denotes calculations by method A, the pink line by method B, the orange line by method C, and the green line by method D. The baseline (BL) calculations by each of the four methods are in the top center axis. One ventilator adjustment was then performed, and the data recalculated. The changes were performed and are listed in counter-clockwise fashion: Vt+ is tidal volume increase by 2 ml/kg of ideal body weight from baseline, Vt- is tidal volume decrease by 2 ml/kg of ideal body weight from baseline, I:E+ is the ratio increase by 1.0 from baseline, I:E- is the ratio decrease by 1.0 from baseline, PEEP+ is PEEP increase by 2 cmH2O from baseline, and PEEP- is PEEP decrease by 2 cmH2O from baseline. Among the ten subjects, methods B and C best approximated one another, and Method A consistently calculated higher compliance and lower driving pressure. Method D did not demonstrate a consistent pattern.

The study aimed to compare compliance and driving pressure measurement methods. Radar plots of the four methods for each subject, under baseline ventilator settings and with each ventilator adjustment, are available in Supplementary Materials 1, 2. A demonstrative plot for one subject is shown in Figure 3. Average compliance and driving pressure measurements, under baseline ventilator settings and with each adjustment, were compared. Average differences between each method were calculated along with 95% confidence intervals and Bland-Altman limits of agreements of these differences; with statistical significance indicated by confidence intervals that do not intersect the zero line (Supplementary Material 3). Method A consistently produced statistically significant higher values of compliance, and lower values of driving pressure, compared to method B and C. Compliance and driving pressure measurements from methods B and C most closely approximated each other; there was no statistically significant difference in method B compared to method C under baseline, volume decrease, I:E increase or decrease, PEEP increase or decrease conditions. There was a statistically significant difference between method B and C under the volume increase condition, with method C on average calculating greater compliance by 2.5 cmH2O (95% CI: −4.7 to −0.3) than Method B and lower driving pressure by 2.04 cmH2O (95% CI: 0.49 to 3.59). Method D did not show a consistent pattern, demonstrating higher measurements of compliance and lower measurements in driving pressure in eight subjects and the reverse findings in two subjects, compared to the other methods.

Linear mixed-effects regression models were used to assess the impact of each ventilator change on each measurement method (Supplementary Material 4). Model summaries show the average linear effect of each ventilator setting change on the compliance or driving pressure outcome; effects greater than 0 indicate that increasing that setting is associated with an increase in the outcome, and decreasing that setting with a decrease in the outcome; and, vice versa for effects less than 0. An increase in I:E resulted in a statistically significant increase in compliance and decrease in driving pressure for methods B and D, with reciprocal changes for a decrease in I:E. An increase in PEEP resulted in a statistically significant decrease in compliance and increase in driving pressure for methods A and B, with reciprocal changes for a decrease in PEEP. However, these values are averages and the patterns did not consistently hold for each individual subject. Tidal volume adjustments had the smallest sample size (*n* = 5) owing to safety limitations. An increase in tidal volume resulted in a statistically significant increase in compliance measurement for methods B, C and D and vice versa. An increase in tidal volume resulted in a statistically significant increase in driving pressure as well in all. Once again, these values were averages and the patterns of tidal volume’s impact on compliance and driving pressure did not consistently hold for each individual subject. This discrepancy where tidal volume has a positive relationship with both compliance and driving pressure is due to the averaging of numerical values with different magnitudes.

Lung severity as assessed by P/F ratio, BMI, paralysis, and use of epoprostenol did not demonstrate any statistically significant impact on the measurements although the small sample size precludes any definitive statement regarding the impact of these variables.

## Discussion

The aim of this study was to evaluate four ways—two quasi-static methods and two dynamic methods—of measuring compliance and driving pressure of the respiratory system. In comparison to the standard method utilizing the P_*plateau*_ minus PEEP (method A), measurements using the inspiratory slope of a constant flow pressure tracing (method C) and using the P_*zf*_ minus PEEP (method B) both demonstrated consistently lower compliance and higher driving pressure calculations. This would suggest that the standard method of measuring compliance and driving pressure, calculated after stress relaxation and gas re-distribution facilitated by the inspiratory hold, may overestimate the compliance and underestimate the driving pressure. Our data, therefore, suggest that utilizing the inspiratory hold may underestimate the risk to the most vulnerable lung units. Current numerical guidance for mechanical ventilation (i.e., peak pressure less than 30 cmH2O, driving pressure less than 15 cmH2O) utilizes the end-inspiratory breath hold and the resulting plateau pressure (Brower et al., 2000; Esteban et al., 2002; Amato et al., 2015). Although the numerical difference in calculations between methods A, B and C may not be dramatic, and the threshold for damage likely differs throughout regions of the heterogenous ARDS lung, these values assume significance if one aims to not surpass such numerical boundaries for safe ventilation. There was no statistically significant difference between compliance and driving pressure averages for methods B and C, except when tidal volume was increased by 2 ml/kg above baseline. Overall, method B and C most closely approximated each other compared to the other methods. P_*zf*_ may therefore be a better quasi-static marker for the maximum pressure experienced by the most vulnerable lung units than P_*plateau*_. Furthermore, for purposes of lung protection, measuring compliance dynamically, utilizing the inspiratory slope during inflation by constant flow ventilation, may be a neglected but more relevant indicator than the static measure we customarily use.

Despite the small total number of subjects, the number of data points collected and the use of each subject’s baseline as a self-control allow for reliable comparisons between methods. The subjects had heterogenous pathology. However, the patterns held true across this heterogenous group. Consequently, our investigation would appear to serve as a valid pilot study for larger future comparisons among these various methods. Both P_*zf*_ and inspiratory slopes are not commonly displayed ventilator outputs. Ventilator manufacturers may find utility incorporating P_*zf*_ outputs during an inspiratory hold. Likewise, although the contour-defining stress index has been incorporated into some ventilator monitoring during constant inspiratory flow, dynamic compliance and driving pressure algorithms based on the inspiratory pressure tracing are not yet available and should rather easily be programmed into any modern ventilator so as to provide relevant, intervention-free, and dynamic breath-by-breath information of additional interest.

The expiratory time constant (method D) did not provide a consistent pattern of compliance and driving pressure measurements, at times greater and at times less than the other methods. Various explanations account for this lack of precision. First, lung deflation typically is not uniexponential (Hamahata et al., 2020). There are therefore multiple time constants that apply during different phases of the expiratory cycle. Second, the speed of lung deflation is impacted by the opening of the expiratory valve and the decompression of the ventilator circuit, which further skews the data. Finally, energy can be lost between inspiration and expiration, known as lung hysteresis (Marini, 2020). As such, measures of the mechanics of lung inflation do not necessarily parallel those of deflation. Our data therefore suggest that expiratory time constant, using an average of two data points in the exhalation phase, is unlikely to be a reliable surrogate for the pressures, stress or strain experienced by the respiratory system during inflation. This method may perform better, however, if conducted using the reportedly successful Al-Rawas et al. protocol, where point-by-point values within the 0.10–0.50 s of the expiratory loop, are recorded (Al-Rawas et al., 2013). This, however, would require either a cumbersome recording process or a programmed data acquisition software that records and transfers data from the ventilator on the order of each millisecond—more frequently than currently reported or displayed.

Ventilator settings were changed (tidal volume, I:E ratio and PEEP) within a standard range of typical clinical adjustments. Some changes of settings appeared to cause statistically significant differences among methods regarding the average compliance and driving pressure measurements. It is difficult, however, to draw firm conclusions regarding predictions of machine adjustments for any given subject; the mentioned patterns of averages for machine setting adjustments did not consistently apply to each individual subject, who varied with regard to underlying lung disorder and severity.

There are various limitations to this study. First, due to judicious use of paralytic agents at our institution, most subjects were not paralyzed. However, subjects and ventilator waveforms were evaluated at the bedside by multiple clinicians to reduce the likelihood of actively initiated ventilation. Additionally, our sample size was diverse and small, owing to light sedation of many intensive care patients and difficulty consenting families of the most critically ill. The modest sample size of the study precludes more detailed conclusions from the data that might apply to disease and severity targeted populations. However, the trends discussed provide a compelling need to reevaluate P_*plateau*_ as the standard base for compliance and driving pressure calculations that currently influence bedside decision-making. Furthermore, this study does not directly address which technique for measuring lung mechanics correlates best with VILI risk, which almost certainly varies with lung vulnerability and with the local environment of the alveoli in question. Yet, as all lung protective techniques attempt to reduce the maximum pressure applied to the alveoli, a technique that reveals higher pressure measurements does suggest that some alveoli are subjected to somewhat higher stresses and strains than previously appreciated.

In conclusion, this study serves as a pilot investigation into various methods for measuring tidal compliance and driving pressure. The standard approach utilizing P_*plateau*_ may overestimate the compliance and underestimate the driving pressure during lung inflation. Further studies are needed to assess the impact of titrating driving pressure and compliance using these alternative methods, the influence of ventilator and patient parameters on these methods, and the potential correlation of these methods with clinical outcomes.

## Data Availability Statement

The raw data supporting the conclusions of this article will be made available by the authors, without undue reservation.

## Ethics Statement

The studies involving human participants were reviewed and approved by Regions Hospital Institutional Review Board. The patients/participants provided their written informed consent to participate in this study.

## Author Contributions

PT prepared the manuscript, participated in the study design, performed the data collection, and assisted in the data analysis. MS, DD, and JM designed the study and obtained the institutional review board approval for the project. FE participated in the study design, data collection, and data interpretation. ME performed statistical analysis of the data. JM was the principal investigator involved in all components of the project. All authors contributed to the article and approved the submitted version.

## Author Disclaimer

The content is solely the responsibility of the authors and does not necessarily represent the official views of the National Institutes of Health’s National Center for Advancing Translational Sciences.

## Conflict of Interest

The authors declare that the research was conducted in the absence of any commercial or financial relationships that could be construed as a potential conflict of interest.

## Publisher’s Note

All claims expressed in this article are solely those of the authors and do not necessarily represent those of their affiliated organizations, or those of the publisher, the editors and the reviewers. Any product that may be evaluated in this article, or claim that may be made by its manufacturer, is not guaranteed or endorsed by the publisher.

## Supplementary Material

Supplementary Material 1 Radar plots showing compliance calculations (in mL/cmH20) for each subject. The blue line denotes calculations by method A, the pink line by method B, the orange line by method C, and the green line by method D. For each subject, the baseline (BL) calculations by each of the four methods are in the top center axis. One ventilator adjustment was then performed, and the data recalculated. The changes were performed and are listed in counter-clockwise fashion: Vt+ is tidal volume increase by 2 ml/kg of ideal body weight from baseline, Vt- is tidal volume decrease by 2 ml/kg of ideal body weight from baseline, I:E+ is the ratio increase by 1.0 from baseline, I:E- is the ratio decrease by 1.0 from baseline, PEEP+ is PEEP increase by 2 cmH2O from baseline, and PEEP- is PEEP decrease by 2 cmH2O from baseline. For subject ID #14, a visual representation could not be displayed as data points were collected only for baseline and Vt+. Among the ten subjects, methods B and C best approximated one another, and Method A consistently calculated higher compliance. Method D did not demonstrate a consistent pattern.

Supplementary Material 2 Radar plots showing driving pressure calculations (in cmH2O) for each subject. The blue line denotes calculations by method A, the pink line by method B, the orange line by method C, and the green line by method D. For each subject, the baseline (BL) calculations by each of the four methods are in the top center axis. One ventilator adjustment was then performed, and the data recalculated. The changes were performed and are listed in counter-clockwise fashion: Vt+ is tidal volume increase by 2 ml/kg of ideal body weight from baseline, Vt- is tidal volume decrease by 2 ml/kg of ideal body weight from baseline, I:E+ is the ratio increase by 1.0 from baseline, I:E- is the ratio decrease by 1.0 from baseline, PEEP+ is PEEP increase by 2 cmH2O from baseline, and PEEP- is PEEP decrease by 2 cmH2O from baseline. For subject ID #14, a visual representation could not be displayed as data points were collected only for baseline and Vt+. Among the ten subjects, methods B and C best approximated one another, and Method A consistently calculated lower driving pressure. Method D did not demonstrate a consistent pattern.

Supplementary Material 3 Mean compliance and driving pressure measurements for methods A, B, C, and D under each ventilator setting and the differences between these means for each method under each setting. Ninety five percent confidence intervals for the differences and Bland-Altman limits of agreement (LOA) are calculated. A statistically significant difference is denoted by confidence intervals that do not intersect the “zero line.”

Supplementary Material 4 Linear mixed-effects regression models showing the average linear effect of each ventilator setting change on the compliance or driving pressure outcome; effects greater than 0 indicate that increasing that setting is associated with an increase in the outcome, and decreasing that setting with a decrease in the outcome; and, vice versa for effects less than 0. Although statistically significant patterns were sometimes noted, the values are averages and the patterns typically did not hold for each individual subject.
