# Supplementary figures and images for: Static and Dynamic Measurements of Compliance and Driving Pressure: A Pilot Study
Source: Front Physiol. 2022 Feb 4;13:773010. doi: 10.3389/fphys.2022.773010 (PMC8854783; doi:10.3389/fphys.2022.773010)

ID 1

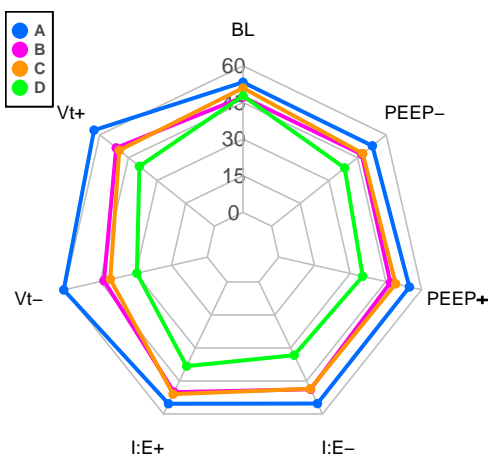

ID 2

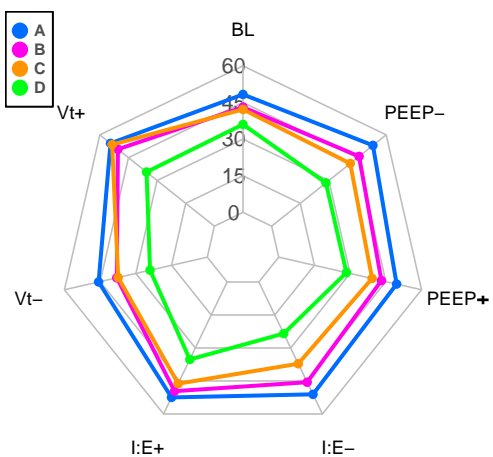

ID 4

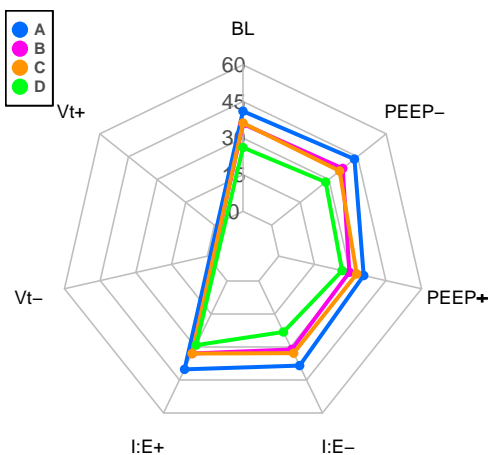

ID 6

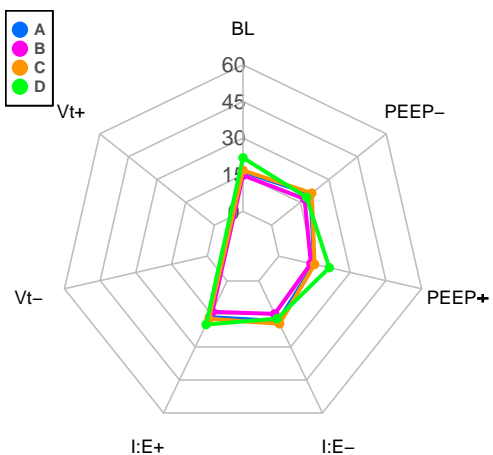

ID 7

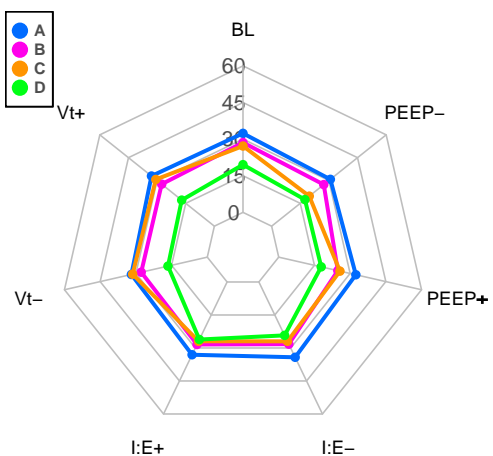

ID 9

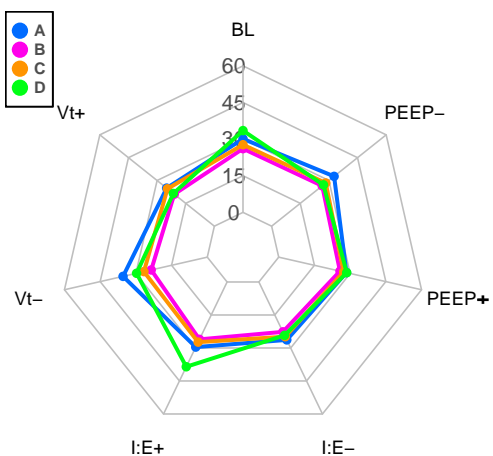

ID 11

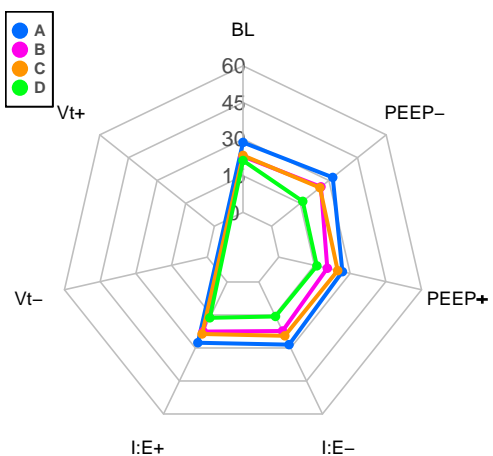

ID 12

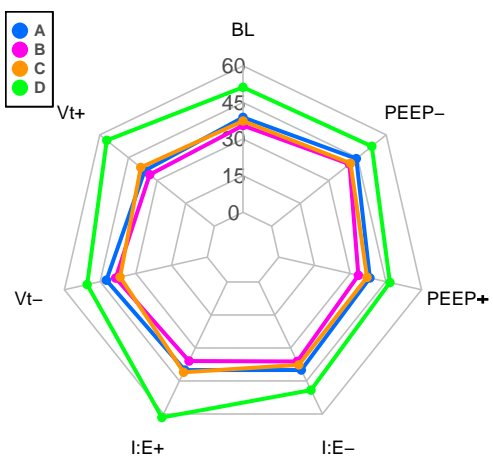

ID 13

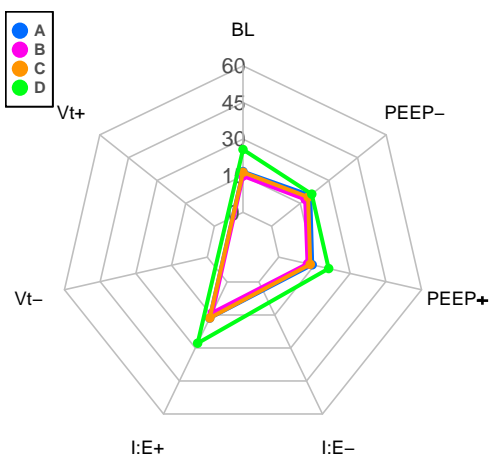

ID 14

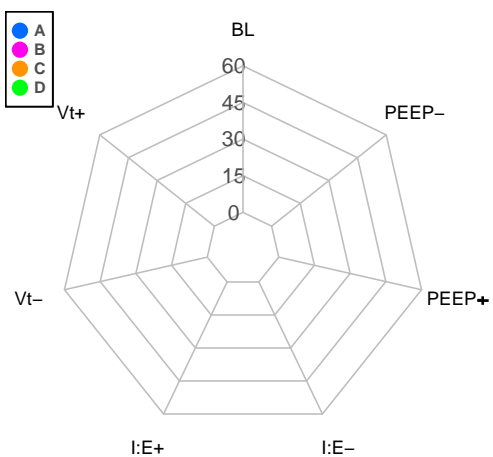

Supplement: Supplementary file 2 [file Image_1.pdf]

**ID 1**

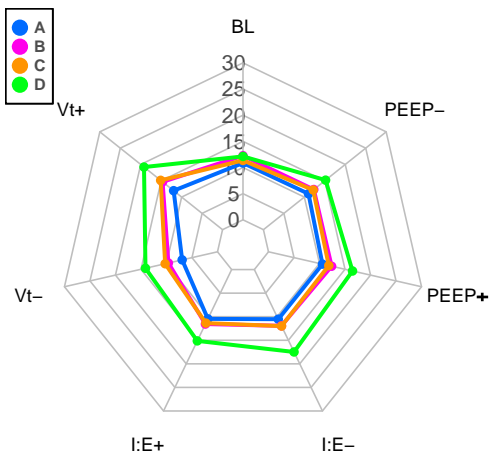

**ID 2**

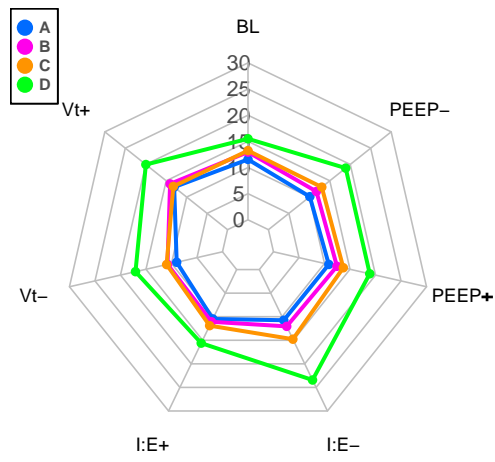

**ID 4**

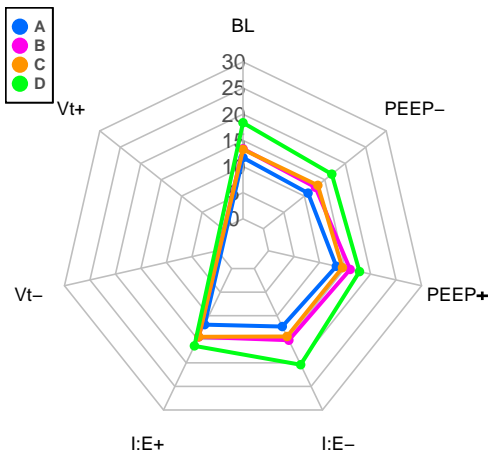

**ID 6**

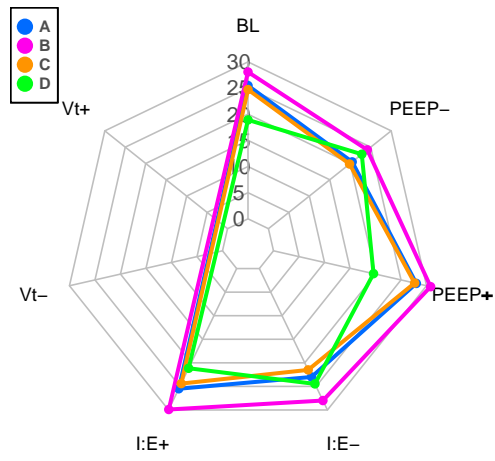

**ID 7**

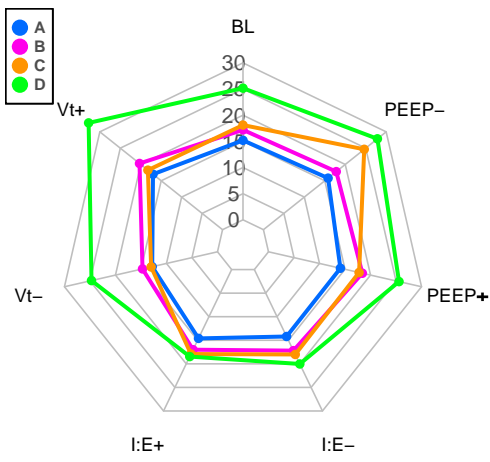

**ID 9**

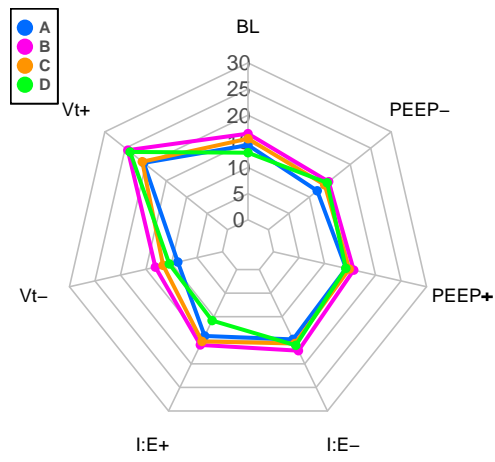

**ID 11**

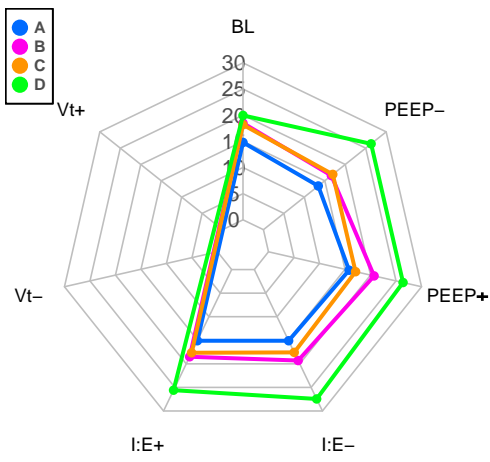

**ID 12**

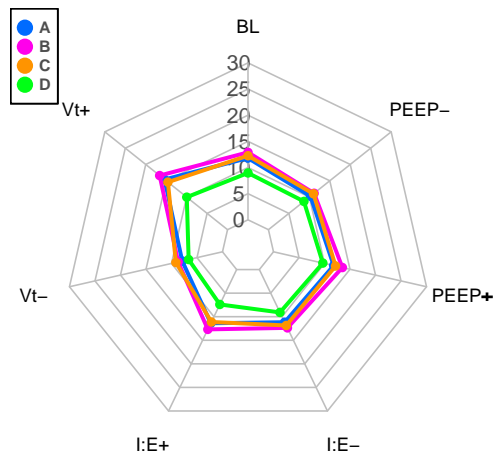

**ID 13**

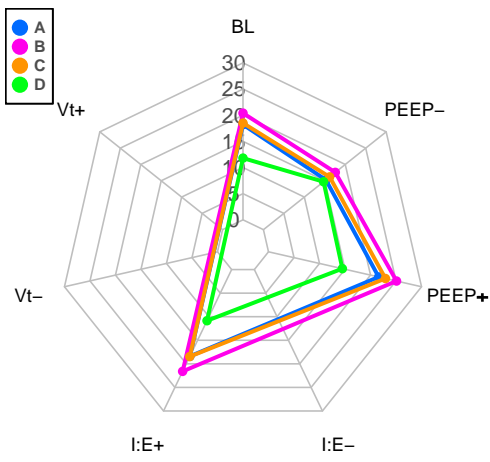

**ID 14**

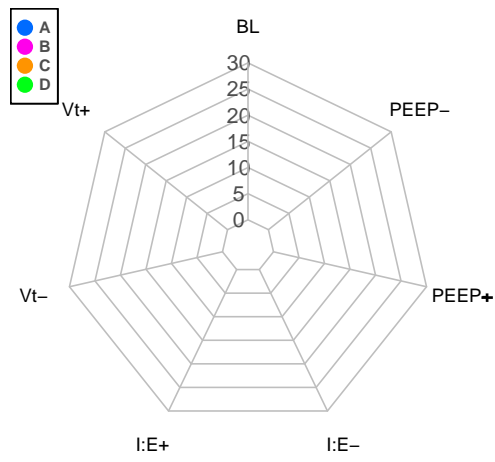

Supplement: Supplementary file 3 [file Image_2.pdf]

## Compliance

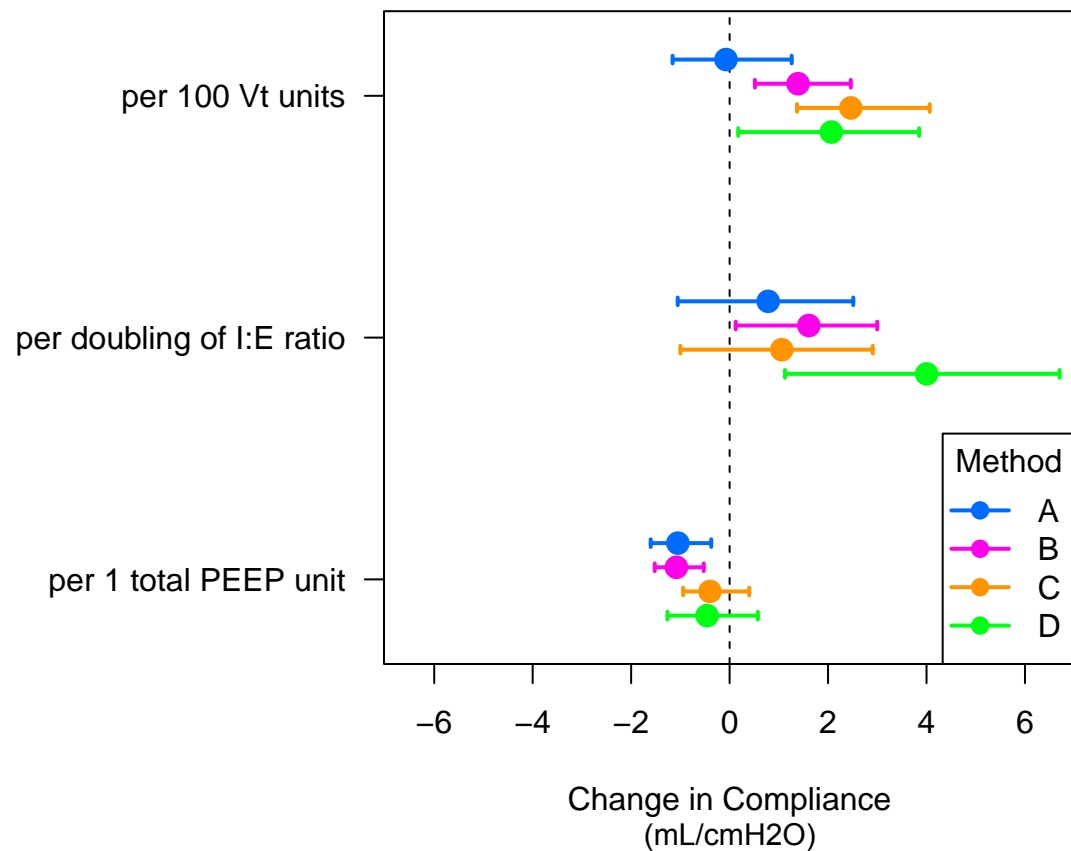

## Driving Pressure

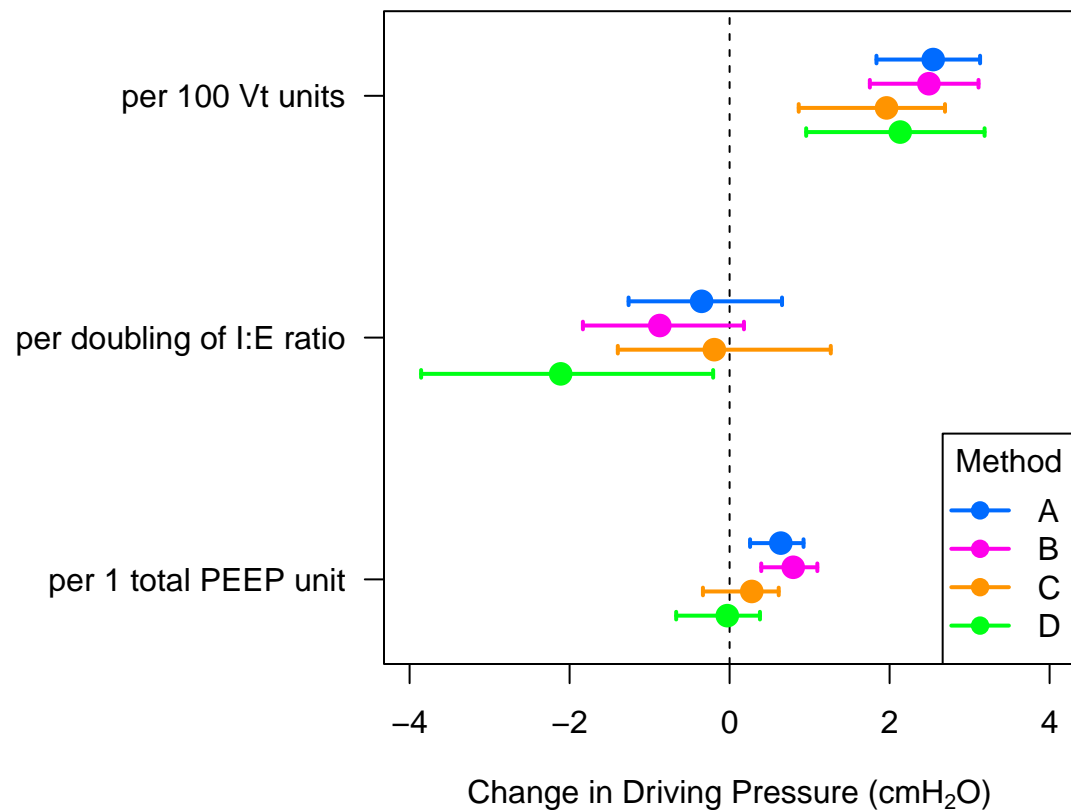

Supplement: Supplementary file 4 [file Image_3.pdf]
